# Tuberculosis, vulnerabilities, and HIV in homeless persons: a systematic review

**DOI:** 10.11606/s1518-8787.2022056003964

**Published:** 2022-05-18

**Authors:** Janaína Rosenburg Gioseffi, Ramaiene Batista, Sandra Mara Brignol

**Affiliations:** I Universidade Federal Fluminense Instituto de Saúde Coletiva Programa de Pós-Graduação em Saúde Coletiva Niterói RJ Brasil Universidade Federal Fluminense. Instituto de Saúde Coletiva. Programa de Pós-Graduação em Saúde Coletiva. Niterói, RJ, Brasil; II Universidade Federal Fluminense Faculdade de Medicina Niterói RJ Brasil Universidade Federal Fluminense. Faculdade de Medicina. Niterói, RJ, Brasil; III Universidade Federal Fluminense Instituto de Saúde Coletiva Departamento de Epidemiologia e Estatística Niterói RJ Brasil Universidade Federal Fluminense. Instituto de Saúde Coletiva. Departamento de Epidemiologia e Estatística. Niterói, RJ, Brasil

**Keywords:** Homeless Persons, Tuberculosis, HIV Infections, Coinfection, epidemiology, Health Vulnerability, Social Vulnerability

## Abstract

**OBJECTIVE:**

Analyze, systematize, and compile social, individual, and programmatic vulnerability factors associated with tuberculosis and HIV in homeless persons.

**METHODS:**

This is a systematic literature review assessing quantitative studies, published between 2014 and 2020, on the prevalence of tuberculosis in homeless persons. Our review grouped studies according to vulnerabilities, followed the PRISMA recommendation guide, and used the Joanna Briggs Institute Critical Appraisal tool for bias analysis.

**RESULTS:**

Of the 372 publications found, 16 were selected according to our eligibility criteria. In total, 10 studies assessed tuberculosis and HIV. The most commonly described factors for individual, social, and programmatic vulnerability were drug use, HIV coinfection, and tuberculosis treatment failure, respectively. The literature also claims that average homelessness length related to a higher frequency of tuberculosis and latent tuberculosis infection.

**CONCLUSION:**

All reviewed studies described how homeless persons suffer with stigma and dehumanization, which are important barriers to their access to health services. Homelessness enhances the risks of chronic and infectious diseases and prioritizes issues which are more pragmatic for the maintenance of life, such as safety and food, to the detriment of health. The results can be used to support hypotheses for future research and to reinforce and direct existing public health and social policies to cope with tuberculosis and HIV in homeless persons.

## INTRODUCTION

Tuberculosis is a respiratory disease caused by the agent *Mycobacterium tuberculosis* . It is currently among the 10 most lethal diseases in the world and the first, among infectious diseases^1^. Its transmission occurs by inhalation of aerosols leading to a granulomatous infection in the lower respiratory tract^2^. Its occurrence is associated with socioeconomic factors since, according to the UN, 95% of cases occur in low- and middle-income countries^3^. Africa and the Americas^1^ are at the top of the ranking of estimated cases and deaths for the disease.

The global estimate of tuberculosis infection was 10 million cases in 2020, with an estimated incidence of 132/100,000 inhabitants, and 1.2 million deaths, of which 208,000 among people who are HIV+^1^. The END TB strategy, proposed by the World Health Organization, aims to reduce the incidence of tuberculosis to less than 10/100,000 inhabitants and decrease the number of deaths by at least 95% so tuberculosis could no longer be considered a global public health problem^4^.

In Brazil, tuberculosis is an important health problem, with a death rate of 2.2/100,000 and an incidence of 31.6/100,000 inhabitants^5^. The Sistema Único de Saúde ( *SUS - Brazilian Unified Health System* ) promotes specific programs to combat it, such as the *Programa Nacional de Controle da Tuberculose* (National Tuberculosis Control Program) and the establishment of treatment directly managed by the basic health network^6^. However, patient adherence is low^7^, and treatment abandonment and wrong or intermittent medication administration cause the number of deaths to remain high and the emergence of drug-resistant strands^7^.

Homeless persons (HP) are constantly exposed to different types of vulnerable and degrading living conditions, increasing the challenge for health care and requiring specific interventions for these people^4 , 8^. As a result of this exposure and the precarization of their lives, tuberculosis in HP is very frequent. This population is 56 times more likely to be affected by this disease in Brazil.

This vulnerabilization has three dimensions^9^: 1) Individual – determined by individuals’ access to information; their ability to put it into practice; and material, cultural, cognitive, and moral aspects – among many others participating in the construction of the human “being”; 2) Social – guided by social and cultural contexts and 3) programmatic – scenarios concerning social institutions (especially health, education, culture, and social assistance) which enable unfavorable contexts to increase these social conditions.

Treating tuberculosis in HP is more expensive and complex than in the rest of the population due to their lower treatment adherence, according to the Evidence Informed Policy Network of the Brazilian Ministry of Health^4^. Moreover, issues such as safety, food, and rest, compete in importance with health care. In addition to tuberculosis, HIV/Aids, dermatological diseases (including leprosy), and hypertension are the main diseases in this population. Psychosocial treatment due to drug and alcohol abuse is also prominent^10^. Homeless persons live marginalized and distant from public policies and lack the effective exercise of their basic rights, including access to medical primary care in SUS^11^.

Worldwide, tuberculosis is the leading cause of death in HIV-positive individuals, accounting for one third of their deaths^12^. People with HIV are 28 times more likely to become infected with tuberculosis^13^, a coinfection responsible for almost 29% of deaths from tuberculosis in Brazil^14^. In 2018, he incidence of tuberculosis in HIV-positive patients was 5.2/100,000 inhabitants in Brazil. In the same year, of the new cases of tuberculosis, 75.5% were tested for HIV and, among the affected, only 47.4% underwent antiretroviral treatment concomitantly with tuberculosis treatment^15^.

Due to the technological advances in tuberculosis and HIV/Aids treatment and the commitments made by World Health Organization member countries, this scenario is problematic and challenging since these diseases are historically important^16^. Fostering knowledge and debate on the tuberculosis epidemic, which is related to social, individual, and programmatic vulnerability in homeless persons, is fundamental to cope with these diseases in Brazil, especially due to the current covid-19 epidemic, in which healthcare struggles to meet different demands. This study aimed to analyze, systematize, and compile the individual, social, and programmatic vulnerability factors associated with tuberculosis and tuberculosis+HIV collected from studies on these illnesses in homeless persons which were conducted between 2014 and 2020.

## METHODS

A systematic literature review was conducted with studies, published from 2014 to 2020, assessing tuberculosis and tuberculosis+HIV in homeless persons. A systematic review is a research method which, in health, consists of seeking and selecting, evaluating, compiling, and showing the published evidence on an important topic and its impact on populations’ health. Preferred Reporting Items for Systematic Reviews and Meta-Analyses (Prisma) criteria were applied.

### Survey Strategy

References were surveyed by the descriptors Tuberculosis, Homeless Persons, HIV, Social Vulnerability, and Health Vulnerability (and their combinations) in the PubMed and Latin American and Caribbean Health Sciences Literature (Lilacs) platforms, as shown in Table 1 [Table t2] . Articles were analyzed by two researchers at two different moments and the results, compiled.

**Table 1:** Search strategies.

Platform	Descriptors	Results
Pubmed http://www.ncbi.nlm.nih.gov/pub med	*(“homeless persons”[MeSH Major Topic] AND “tuberculosis”[MeSH Major Topic]) AND “hiv”[MeSH]*	4
Pubmed http://www.ncbi.nlm.nih.gov/pub med	*(“tuberculosis”[MeSH Major Topic]) AND “homeless persons”[MeSH Major Topic]*	229
Pubmed http://www.ncbi.nlm.nih.gov/pub med	*((“Tuberculosis/analysis”[Mesh] OR “Tuberculosis/epidemiology”[Mesh] OR “Tuberculosis/mortality”[Mesh] OR “Tuberculosis/statistics and numerical data”[Mesh] OR “Tuberculosis/transmission”[Mesh])) AND (“Homeless Persons/epidemiology”[Mesh] OR “Homeless Persons/mortality”[Mesh] OR “Homeless Persons/statistics and numerical data”[Mesh])*	104
Lilacs http://lilacs.bvsalud.org/en/	*tuberculose [Descritor de assunto] AND pessoas em situação de rua [Descritor de assunto]*	13
Lilacs http://lilacs.bvsalud.org/en/	*pessoas em situação de rua [Descritor de assunto] AND vulnerabilidade social [Descritor de assunto],*	
Lilacs http://lilacs.bvsalud.org/en/	*pessoas em situação de rua [Descritor de assunto] AND vulnerabilidade social [Descritor de assunto] AND tuberculose [Descritor de assunto]*	1
Lilacs http://lilacs.bvsalud.org/en/	*vulnerabilidade social [Descritor de assunto] AND tuberculose [Descritor de assunto]*	5
Lilacs http://lilacs.bvsalud.org/en/	*vulnerabilidade em saúde [Descritor de assunto] AND tuberculose [Descritor de assunto]*	5

**Table 2 t2:** Descriptors and articles selected from the studies.

Authors	Study population	Year	Country	Descriptors	Study Design
Aldridge et al.^21^	491 homeless persons	2018	England	Tuberculosis + Homeless persons	Cross-sectional study
Semunigus et al.^22^	351 homeless persons	2016	Ethiopia	Tuberculosis + Homeless persons + HIV	Cross-sectional study
Vieira et al.^23^	Population of 18 Portuguese districts	2018	Portugal	Tuberculosis + Homeless people	Ecological study
Ranzani et al.^24^	1,726 homeless persons	2016	Brazil	Tuberculosis + Homeless persons	Cohort study
Hwang et al.^25^	3,292 homeless persons	2017	South Korea	Tuberculosis + Homeless persons	Cohort study
Dias et al.^26^	92,053 homeless persons	2017	Portugal	Tuberculosis + Homeless persons	Cohort study
Agarwal et al.^27^	543 homeless persons	2019	USA	Tuberculosis + Homeless persons	Cross-sectional study
Nwana et al.^28^	393 homeless persons	2019	USA	Tuberculosis + Homeless persons	Cohort study
Amiri et al.^29^	593 homeless persons	2014	Iran	Tuberculosis + Homeless persons	Cross-sectional study
Powell et al.^30^	110 occurrences of multidrug-resistant tuberculosis	2017	USA	Tuberculosis + Homeless persons	Cross-sectional study
Gomez et al.^31^	544 homeless persons with tuberculosis	2019	Colombia	Tuberculosis + Homeless persons	Cohort study
Munn et al.^32^	64 homeless persons	2015	USA	Tuberculosis + Homeless persons	Cohort study
Streit et al.^33^	142 homeless persons	2019	Germany	Tuberculosis + Homeless persons	Cross-sectional study
Dolla et al.^34^	301 homeless persons	2017	India	Tuberculosis + Homeless persons	Cross-sectional study
Korzeniewska-Koseła et al.^35^	2,349 homeless persons	2015	Poland	Tuberculosis + Homeless persons	Cohort study
Pendzich et al.^36^	117 homeless persons	2015	Poland	Tuberculosis + Homeless persons	Cohort study

### Eligibility Criteria

Analyses included studies with a quantitative design, written in English, Spanish, and Portuguese and published from 2014 to 2020, which assessed tuberculosis, tuberculosis+HIV, and the vulnerability factors associated with these illnesses.

### Bias Analysis

Bias in the evaluated studies was assessed in pairs, using the Joanna Briggs Institute Critical Appraisal (JBI – Systematic Reviews tools). JBI is composed of questions assessing the methodological quality of a study according to its design. Cross-sectional studies were evaluated with the JBI Critical Appraisal Checklist for Analytical Cross Sectional Studies with the following questions: (i) clearly defined inclusion and exclusion criteria; (ii) theme and method described in detail; (iii) appropriately measured exposures; (iv) objective and standardized definition criteria to determine the condition studied; (v) confounding factor identification; (vi) strategies to deal with confounding factors; (vii) adequately measured outcomes; and (viii) appropriate statistical analyses^18^. To analyze cohort studies, 11 questions of the JBI Critical Appraisal Checklist for Analytical Cohort Studies were used: (i) both groups recruited from the same population; (ii) exposures similarly measured to identify exposed and unexposed groups; (iii) appropriately measured exposures; (iv) confounding factor identification; (v) strategies for dealing with confounding factors; (vi) participants free of outcomes at baseline; (vii) appropriately measured outcomes; (viii) sufficient time to study outcome occurrence; (ix) complete follow-ups/if not, described and explored reasons for it; (x) strategies to deal with incomplete follow-ups; and (xi) appropriate statistical analyses^19^. Ecological studies were evaluated by the JBI Critical Appraisal Checklist for Analytical Cross Sectional Studies, modified according to criteria in Dufault and Klar^20^for the methodological evaluation of this type of study design, with the following questions: (i) explained chosen design and sample size; (ii) clearly defined inclusion and exclusion criteria; (iii) theme and method described in detail; (iv) objective and standardized definition criteria to determine the condition studied; (v) appropriately measured exposures; (vi) confounding factor identification; (vii) strategies to deal with confounding factors; (viii) appropriately measured outcomes; (ix) efforts to reduce the possibility of bias; (x) appropriate statistical analyses; (xi) strategies to deal with incomplete follow-ups; and (xii) highlighted limitations^18 , 20^.

## RESULTS

We found 374 studies in the researched platforms, selecting 103 published within the study period and excluding two since they were official government documents for public policies, 23 for being duplicates, and nine because they were not indexed for full reading. Finally, 71 studies remained for analysis. We read their abstracts and browsed the entire articles, selecting 16 according to our eligibility criteria. Most studies were conducted in Europe (6/16), followed by Asia (3/16), North America (4/16), South America (2/16) – of which one was Brazilian, and Africa (1/16) ( Figure ). In total, seven were cross-sectional studies; eight, cohort; and one, ecological. To this, we added the average of 2.7 studies published annually in the years analyzed, with a minimum of one and a maximum of four papers published each year.

**Figure:**
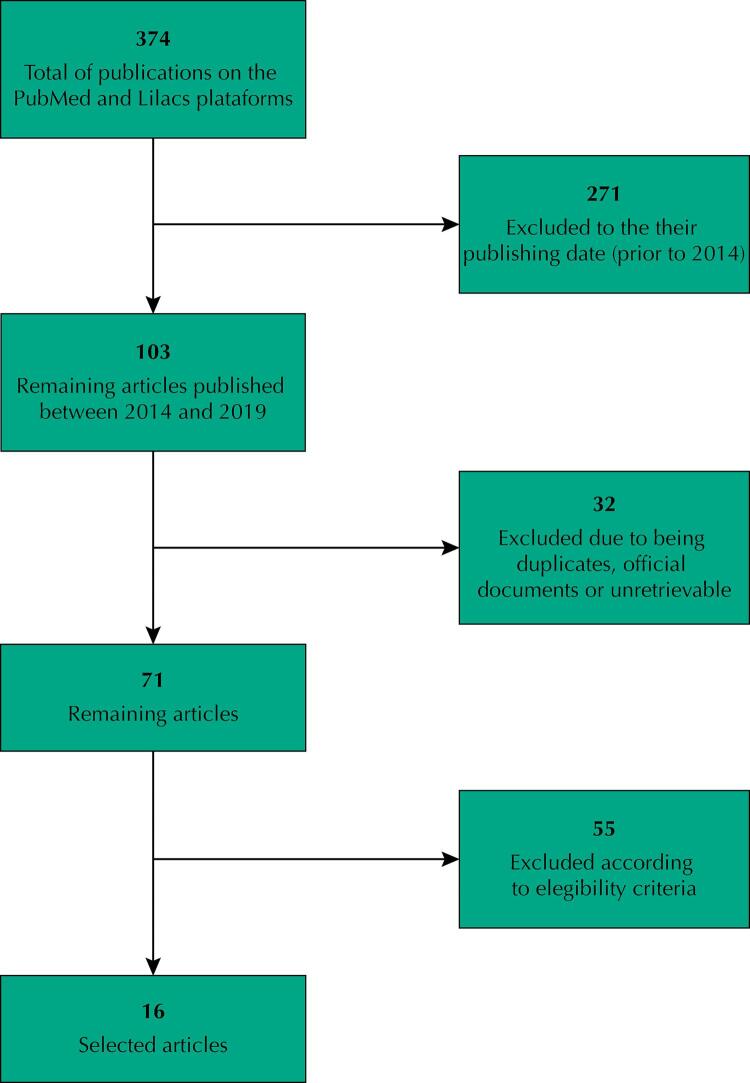
Result flowchart.

We collected our sample between June 2019 and April 2021 and all data were entered into a Microsoft Excel spreadsheet containing the authors, study population, year of publication, country of publication, and study design.

Among the selected articles, we found the following vulnerability factors for their outcomes: alcohol use^21^; smoking^21 , 22 , 25 , 28 , 29^; use of illicit drugs^21 , 24 , 26^, such as injectable ones^21 , 27^ - which may have caused needle sharing^29 , 30^ - and methamphetamine^29^; history of incarceration^21 , 29^; immigration^26 , 32 , 33^; psychological disorders^24 , 28 , 30^; prostitution^29^, including among men who have sex with men; illiteracy^22 , 29^; malnutrition^22^; HIV coinfection^21 , 22 , 24^; and other chronic diseases, such as diabetes^27 , 28^, chronic renal failure^27 , 28^, and hepatitis B and C^21 , 28 , 29^.

In all analyzed studies, most patients with tuberculosis were Black or mixed-race men^24 , 28 , 30 , 32^, with a mean age of 49.8 years (SD ± 5.2). The literature shows that average homelessness length related to a higher occurrence of tuberculosis and latent tuberculosis infection^21 , 22 , 29^.

Table 3 [Table t4] shows the outcomes related to the occurrence of tuberculosis and latent tuberculosis infection: incomplete treatment^22 , 24 , 25 , 27 , 28 , 30 , 31 , 34 , 35^ or its failure^2 , 24 , 26^, death^22 , 24 , 25 , 27 , 30 , 31 , 34^, development of treatment-resistance^22 , 25^, and occurrence of extrapulmonary tuberculosis^22 , 26 , 27^. The main diagnostic tools were sputum cytology^22 , 24 , 26 , 27 , 29 , 31^, thoracic X-rays^24 , 27 , 33^, culture^27 , 29^, molecular examination^27^, and clinical diagnosis^27^.

**Table 3:** Summarization of vulnerability factors.

Vulnerability factor	Vulnerability dimension	Author
Psychological disorder	Individual	Ranzani et al.^24^ (2016); Nwana et al.^28^ (2019); Powell et al.^30^ (2017)
Alcohol use	Individual and social	Aldridge et al.^21^ (2018); Semunigus et al.^22^ (2016); Vieira et al.^23^ (2018); Ranzani et al.^24^ (2016); Hwuang et al.^25^ (2017); Dias et al.^26^ (2017); Agarwal et al.^27^ (2019); Nwana et al.^28^ (2019)
Tobacco use	Individual and social	Aldridge et al.^21^ (2018); Semunigus et al.^22^ (2016); Hwuang et al.^25^ (2017); Nwana et al.^28^ (2019) Amiri et al.^29^(2014)
Drug use	Individual and social	Aldridge et al.^21 ^(2018); Ranzani et al.^24^ (2016); Dias et al.^26^ (2017); Agarwal et al.^27^ (2019); Nwana et al.^28^ (2019); Powell et al.^30^ (2017)
Low educational attainment	Individual and social	Semunigus et al.^22^ (2016); Ranzani et al.^24^ (2016); Amiri et al.^29^ (2014)
Immigration	Social	Dias et al.^26^ (2017); Munn et al.^32^ (2015); Streit et al.^33^ (2019)
Imprisonment	Social	Aldridge et al.^21^ (2018); Amiri et al.^29^ (2014); Powell et al.^30^ (2017)
Engaging in illegal activities (theft, drug trafficking, and prostitution)	Social	Amiri et al.^29^ (2014)
Malnutrition	Social	Semunigus et al.^22^ (2016)
Coinfection with HIV and other diseases such as hypertension, hepatitis, and syphilis, as well as tuberculosis recurrence	Social	Aldridge et al.^21^ (2018); Semunigus et al.^22^ (2016); Ranzani et al.^24^ (2016); Hwuang et al.^25^ (2017); Dias et al.^26^ (2017); Agarwal et al.^27^ (2019); Nwana et al.^28^ (2019); Amiri et al.^29^ (2014)
Mixed-race and Black people	Social	Powell et al.^30^ (2017); Munn et al.^32^ (2015); Dolla et al.^34^ (2017)
Treatment failure	Programmatic	Semunigus et al.^22^ (2016); Ranzani et al.^24^ (2016); Hwuang et al.^25^ (2017); Dias et al.^26^ (2017); Agarwal et al.^27^ (2019); Nwana et al.^28^ (2019); Gomez et al.^31^ (2019); Streit et al.^33^ (2019); Dolla et al.^34^ (2017); Korzeniewska-Koseła et al.^35^ (2015)

**Table 4 t4:** Incidences and prevalence found in the reviewed studies.

Authors (year)	Tuberculosis incidence	Tuberculosis prevalence	Latent tuberculosis infection prevalence	Tuberculosis+HIV prevalence	Latent tuberculosis infection+HIV prevalence
Aldridge et al.^21^ (2018)			16,50%		
Semunigus et al.^22^ (2016)	505/100,000 inhabitants	2,60%		55,50%	
Ranzani et al.^24^ (2016)		2,80%		17,30%	
Hwuang et al.^25^ (2017)				5,70%	
Dias et al.^26^ (2017)	122/100,000 inhabitants			32,60%	
Agarwal et al.^27^ (2019)		4,10%		16%	
Nwana et al.^28^ (2019)					1,80%
Amiri et al.^29^ (2014)			46,70%		1,20%
Gomez et al.^31^ (2019)				20,60%	
Streit et al.^33^ (2019			16%		
Dolla et al.^34^ (2017)	270/100,000 inhabitants			20%	

Among the 16 publications analyzed, 10 described the association between tuberculosis comorbidities and HIV. In Semunigus et al.^22^, the proportion of coinfection reached 55.5%. According to Dias et al.^26^, HIV-positive people were 2.1 times more likely to have an unfavorable outcome. Moreover, 32.6% of participants showed some comorbidity. In Ranzani et al.^24^, 17.3% of homeless persons were HIV positive. According to Agarwal et al.^27^, 16% of participants with tuberculosis were HIV positive and the chance ratio for mortality in these individuals was 3.57 higher than for those who were HIV negative. In Hwang et al.^25^, 5.7% showed tuberculosis+HIV coinfections. In Amiri et al.^29^, 1.2% of HP were HIV positive and had a latent tuberculosis infection, a similar proportion (1.8%) to the one in Nwana et al.^28^ Gomez et al.^31^found that 20.6% of HP had comorbidities. Vieira et al.^23^ report that a 100-case increase of HIV+tuberculosis coinfection in the general population raises the incidence of tuberculosis among homeless persons to 14 cases per 100,000 inhabitants. The only HIV+ patient in Dolla et al.^34^ died.

Regarding biases, most studies showed dissimilar recruited groups^24 , 25 , 36^, confounding factor identification^26 , 27 , 29 , 30 , 33^, strategies to deal with these factors^26 , 27 , 29 , 30 , 32^or incomplete participant follow-ups^25 , 26 , 36^. Few studies indicated their limitations^24 , 26 , 28 , 29 , 31 , 34^ and those that did showed flaws in how they described their methodology and results^29 , 30 , 32 , 36^.

## DISCUSSION

We can observe a recurrence between alcohol, tobacco, and illicit drug consumption results – factors of individual susceptibility and social vulnerability (due to the stigma associated with addiction) – whether to escape the reality of suffering or to seek an improvement in general well-being^37 , 38^. Thus, this vulnerability overlap may increase exposure to tuberculosis and HIV in the study population. Gender, age, coinfection by other communicable diseases, and history of incarceration are also factors which arouse discrimination in our society, albeit at different levels.

Since it is a very important global public health problem (even listed among the Sustainable Development Goals), some methodological flaws in these studies draw our attention, such as their small sample size, weakening their results and conclusions; lack of direct access to tuberculosis-positive patients^29^; underestimation of the actual number of homeless persons^24 , 26^ and of the incidence and prevalence of tuberculosis in this population^26 , 34^; and a sample composed only of people with tuberculosis^24^, excluding extrapulmonary forms of the illness.

### Individual Vulnerability

Constant drug use can lead to the development of mental disorders, as stated in the last World Drug Report in 2020 from the United Nations Office of Drugs and Crime (UNODC). In total, two publications^24 , 30^ described the variable “mental disorder,” evincing its constant occurrence in the study samples. The break with family members either due to maladaptation to their structural model, a history of violence and harassment or unacceptance of reprehensible forms of sustenance and their own addiction, expose these people to social and individual vulnerability since they are unable to count on their families at a difficult time^39 , 40^.

### Social Vulnerability

The State attempts to reduce social vulnerability by implementing income transfer programs to decrease the number of people below the poverty line and give them better feeding conditions^41^. This important measure is considered programmatic and affects social vulnerability and should be expanded as it can reduce the vulnerability of people below the poverty line. Since vulnerability dimensions may overlap or intersect, this helps to broaden the epidemiological view on the ways of getting sick and alert managers to health issues, resulting in a more qualified confrontation of these problems in HP.

Producing vulnerable and non-integrable lives forces HP to continuously live with access barriers to education, work, and health care and services, among others, in addition to their invisibility, which can lead some of them to engage in activities such as prostitution, association with trafficking, theft, and labor analogous to slavery.

Social vulnerability also involves characteristics marked by lower social classes, residence, and Black ethnicity, among others. Studies showed a 63.6% proportion of Black and mixed-race people among their results. The average age of participants is 49.8 years (SD ± 5.2), probably due to their greater spatial mobility, when compared to women or people of different age groups such as older adults, who move less through the streets, exposing themselves less in their journeys^42^.

History of incarceration was frequent among the reviewed studies. The prison system, tasked with rehabilitating and reinserting convicts into society, fails to do so due to the stigma inmates carry after their sentences are fulfilled either by society in general or by absent family support or unemployment. Thus, many lack the monetary resources to continue their lives, resorting to homelessness and sometimes engaging in illegal activities, which are incapable of breaking the recidivism cycle^43^.

According to a ranking conducted by the World Prison Brief (2021)^44^, Brazil has the third largest prison population in the world, a fact intrinsically linked to structural racism and, consequently, to the systematic marginalization of Black and mixed-race people, who comprise 61.7% of inmates^45^. Thus, we can observe that these vulnerability factors related to social, racial, and economic impacts are perpetuated in the most vulnerable populations.

### Programmatic Vulnerability

This dimension, related to access to State public and institutional equipment, proved to be an important dimension to analyze vulnerable populations’ susceptibilities to tuberculosis and HIV in epidemiological studies^46^, as it evinces the precariousness and fragility of these resources. Incomplete treatment and the development of resistance to medication are included in this dimension, as they show the failure of the health system to provide adequate therapy, information, and structure to treat these patients^47^. Among its consequences is coinfection with other diseases since homelessness and environmental exposure enhance the risks for some chronic and infectious diseases^38^ once priority issues for the maintenance of life, such as safety and food, are more urgent than health care. Due to access barriers, health care is relegated to the background or received only when opportune^38 , 48^.

## CONCLUSION

According to the Institute of Applied Economic Research, Brazil had more than 100,000 homeless persons in 2016^49^. Due to the Brazilian socioeconomic perspective, including the covid-19 pandemic allied to the current economic and political scenario, Brazil witnessed an increase in poverty and the number of HP^50^. Among its consequences, we can observe, in the short term, an increase of tuberculosis in HP and other vulnerable populations, such as low-income families, *favela* dwellers or residents of precarious housing^51^ since Brazil already has experienced an increase in poverty and misery, as have other Latin American countries^50 , 52^.

Our perspective of the concept of vulnerability allowed us to assess that the results found in the analyzed publications evinced the social and programmatic factors associated with tuberculosis and HIV in HP, highlighting the different dimensions of life and healthcare precarization. Thus, this concept was a fundamental support to classify and understand the different susceptibilities permeating HP’s life and hindering the treatment of tuberculosis and HIV, as well as treatment failures due to discontinuity and, consequently, the appearance of drug-resistant strains.

Even with established protocols, guidelines, ordinances, and technical standards to prevent and treat HP’s health, we noticed that the execution of the action plans outlined by government strategies shows important flaws, evincing weaknesses in the health system and public policies aimed at HP, and the stigma and social prejudices health services reproduce. Thus, some lives are more exposed to vulnerability situations and consequently show less access to prevention and protection and are more susceptible to violence from the State and society than others. This is due to various crossings marking these lives as more vulnerable and often unrecognized and invisible.

### Recommendations

Based on the results of this review, we recommend greater attention and investment in improving prevention actions and interventions for tuberculosis and tuberculosis+HIV in HP since primary care to routinely assess homeless persons’ vulnerabilities. We also suggest that managers and technicians evaluate the need to change and adjust primary care protocols for HP and integrally distribute resources to the demands which are already known and shown in this study. Political effort and commitment are also necessary to distribute resources destined to health, social policies, and HP assistance.

Systematizing vulnerability factors can support hypotheses for future research and subsidize public health and social policies to cope with tuberculosis and HIV in HP. Our findings, when compared to other systematic reviews, seem to advance the literature by its discussion from a perspective of the concept of vulnerability, unprecedented in national publications, for homeless persons and the prevalence of tuberculosis and tuberculosis+HIV.

### Limitations

This study has limitations since, even if its two researchers conducted a wide search of the published studies, a number of texts may yet be digitally unavailable on the surveyed platforms; due to the quick dynamic of publishing, some studies may have escaped search and data collection.
