# Supplementary material for: Tuberculosis, vulnerabilities, and HIV in homeless persons: a systematic review
Source: Rev Saude Publica. 2022 May 18;56:43. doi: 10.11606/s1518-8787.2022056003964 (PMC9126575; doi:10.11606/s1518-8787.2022056003964)
Supplement: Translation

# Translation: Tuberculose, vulnerabilidades e HIV em pessoas em situação de rua: revisão sistemática

**Authors:** Janaína Rosenburg Gioseffi, Ramaiene Batista, Sandra Mara Brignol

**Affiliations:** I Brasil Universidade Federal Fluminense. Instituto de Saúde Coletiva. Programa de Pós-Graduação em Saúde Coletiva. Niterói, RJ, Brasil; II Brasil Universidade Federal Fluminense. Faculdade de Medicina. Niterói, RJ, Brasil; III Brasil Universidade Federal Fluminense. Instituto de Saúde Coletiva. Departamento de Epidemiologia e Estatística. Niterói, RJ, Brasil

**Keywords:** Pessoas em Situação de Rua, Tuberculose, Infecções por HIV, Coinfecção, epidemiologia, Vulnerabilidade em Saúde, Vulnerabilidade Social

## Abstract

**OBJETIVO:**

Analisar, sistematizar e compilar os fatores de vulnerabilidade (social, individual e programática) associados à tuberculose e HIV em pessoas em situação de rua.

**MÉTODOS:**

Revisão sistemática de literatura de publicações quantitativas sobre tuberculose em pessoas em situação de rua entre os anos de 2014 e 2020, seguindo o guia de recomendações PRISMA e, para análise de viés, a ferramenta *Joanna Briggs Institute Critical Appraisal.* Agrupou-se as publicações segundo as vulnerabilidades.

**RESULTADOS:**

Entre 372 publicações encontradas, selecionaram-se 16 segundo os critérios de elegibilidade. Em 10 estudos, foi descrita ocorrência de tuberculose e HIV. Os fatores de vulnerabilidade individual, social e programática mais descritas foram uso de drogas, coinfecção com HIV e falha no tratamento da tuberculose, respectivamente. A média de tempo em situação de rua também se mostrou relacionada à maior frequência de tuberculose e da infecção latente da tuberculose segundo literatura.

**CONCLUSÃO:**

O estigma e a desumanização associados às pessoas em situação de rua foram descritos em todos os estudos revisados, sendo importantes barreiras no acesso aos serviços de saúde. A vivência na rua potencializa os riscos para a ocorrência de doenças crônicas e infecciosas, bem como a priorização de questões mais pragmáticas à manutenção da vida, como segurança e alimentação, em detrimento à saúde. Os resultados encontrados podem ser utilizados para embasar hipóteses para futuras pesquisas e para reforçar e direcionar políticas públicas de saúde e sociais já existentes para o enfrentamento da tuberculose e HIV na pessoa em situação de rua.

## INTRODUÇÃO

A tuberculose é uma doença respiratória causada pelo agente *Mycobacterium tuberculosis* que está entre as dez doenças que mais matam no mundo atualmente e é a primeira colocada em causas de óbito entre as doenças infecciosas^1^. Sua transmissão ocorre por inalação de aerossóis e conduz a uma infecção granulomatosa em trato respiratório inferior^2^e sua ocorrência está associada a fatores socioeconômicos, visto que, segundo a ONU, 95% dos casos ocorrem em países de média e baixa renda^3^e, no topo do ranking de casos estimados e de mortes para a doença, estão o continente africano e as Américas^1^.

A estimativa global de infecção pela tuberculose em 2020 foi de 10 milhões de casos, com uma incidência estimada de 132/100.000 habitantes, e de 1,2 milhão de mortes no ano, além de 208 mil óbitos entre pessoas HIV+^1^. A estratégia *END TB* , proposta pela Organização Mundial da Saúde, tem como metas diminuir a incidência da tuberculose para menos de 10/100.000 habitantes e reduzir o número de óbitos em pelo menos 95%, dessa forma a tuberculose poderia deixar de ser considerada um problema de saúde pública mundial^4^.

No Brasil, a tuberculose é um importante problema de saúde, com uma taxa de óbitos de 2,2/100.000 habitantes e uma incidência de 31,6/100.000 habitantes^5^e conta com programas específicos do Sistema Único de Saúde (SUS) para seu combate, como o Programa Nacional de Controle da Tuberculose e o estabelecimento do tratamento diretamente observado pela rede básica de saúde^6^. Porém, a adesão dos pacientes é baixa^7^e o abandono do tratamento e a administração errada ou intermitente dos medicamentos fazem com que o número de óbitos permaneça elevado e que se crie resistência aos fármacos utilizados^7^.

As pessoas em situação de rua (PSR) estão constantemente expostas a diferentes tipos de vulnerabilização e condições de vida degradantes, o que aumenta o desafio para o cuidado em saúde, necessitando de intervenções específicas para essas pessoas^4 , 8^. Em consequência dessa exposição e precarização de suas vidas, a tuberculose entre PSR é muito frequente e apresentam 56 vezes mais chances de serem acometidas por esse agravo no Brasil.

Essa vulnerabilização tem três dimensões^9^: 1) Individual – determinada pelo que o indivíduo dispõe de informações, de sua capacidade de pô-las em prática, e de aspectos materiais, culturais, cognitivos e morais, entre tantos outros que participam da construção do “ser” humano; 2) Social – pautada pelos contextos sociais e cenários culturais; 3) Programática - que diz respeito às instituições, principalmente de saúde, educação, cultura e assistência social, permitindo contextos desfavoráveis, incrementando essas condições sociais.

O tratamento da tuberculose em PSR é mais caro e complexo quando comparado ao restante da população, com menor adesão ao tratamento, segundo a Rede para Políticas Informadas por Evidências do Ministério da Saúde do Brasil^4^, questões como segurança, alimentação e descanso competem em importância com o cuidado da saúde na PSR. Além da tuberculose, o HIV/aids, doenças dermatológicas (incluindo hanseníase) e hipertensão arterial são as principais doenças incidentes nessa população, bem como acompanhamento psicossocial devido ao abuso de drogas e álcool^10^. As pessoas em situação de rua vivem marginalizadas e distantes das políticas públicas e sem o efetivo cumprimento de seus direitos básicos, inclusive quando se trata de atendimento médico na atenção básica no SUS^11^.

Em todo o mundo, a tuberculose é a principal causa de morte em HIV positivos, representando um terço das mortes em decorrência da aids^12^. As pessoas HIV positivas têm 28 vezes mais chances de se infectar com a tuberculose^13^e essa coinfecção representa quase 29% das mortes no Brasil^14^. No ano de 2018 no Brasil, a incidência de tuberculose em pacientes HIV-positivos foi de 5,2/100.000 habitantes. No mesmo ano, dos novos casos de tuberculose, 75,5% foram testados para HIV e, dentre os positivos, apenas 47,4% fizeram o tratamento antirretroviral concomitantemente ao tratamento para a tuberculose^15^.

Diante dos avanços tecnológicos nos tratamentos de tuberculose e HIV/aids e dos compromissos assumidos pelos países membros da Organização Mundial da Saúde, esse cenário é problemático e desafiador, visto que essas doenças apresentam uma importância histórica^16^. Fomentar a produção de conhecimento e o debate no contexto da epidemia da tuberculose referente à vulnerabilidade social, individual e programática na população em situação de rua é fundamental para o enfrentamento desses agravos no país, principalmente no contexto atual da epidemia de covid-19, onde a atenção em saúde compete para os atendimentos das diferentes demandas. O objetivo do artigo foi analisar, sistematizar e compilar os fatores de vulnerabilidade (individual, social e programática) associados à tuberculose e tuberculose+HIV, coletados junto aos estudos sobre esses agravos na população das pessoas em situação de rua entre os anos de 2014 e 2020.

## MÉTODOS

Foi realizada uma revisão sistemática de literatura para os agravos de tuberculose e tuberculose+HIV no período de 2014 a 2020 na população em situação de rua. A revisão sistemática é um método de pesquisa que, na área da saúde, consiste em buscar e selecionar, avaliar, compilar e apresentar as evidências publicadas sobre um tema importante e de impacto na saúde das populações. Neste artigo, utilizamos o protocolo *Preferred Reporting Items for Systematic Reviews and Meta-Analyses* (PRISMA)^17^.

### Estratégia de Busca

A busca de referências foi realizada a partir dos descritores *Tuberculosis, Homeless persons, HIV, Social Vulnerability, e Health Vulnerability* e suas combinações entre si nas plataformas de pesquisa PubMed e Literatura Latino-americana e do Caribe em Ciências da Saúde (Lilacs), como pode ser observado na tabela 1 [Table t2002] . As análises dos artigos foram realizadas por duas pesquisadoras em dois momentos distintos e os resultados foram compilados.

**Tabela 1:** Estratégias de busca.

Plataforma	Descritores	Resultados
Pubmed http://www.ncbi.nlm.nih.gov/pub med	*(“homeless persons”[MeSH Major Topic] AND “tuberculosis”[MeSH Major Topic]) AND “hiv”[MeSH]*	4
Pubmed http://www.ncbi.nlm.nih.gov/pub med	*(“tuberculosis”[MeSH Major Topic]) AND “homeless persons”[MeSH Major Topic]*	229
Pubmed http://www.ncbi.nlm.nih.gov/pub med	*((“Tuberculosis/analysis”[Mesh] OR “Tuberculosis/epidemiology”[Mesh] OR “Tuberculosis/mortality”[Mesh] OR “Tuberculosis/statistics and numerical data”[Mesh] OR “Tuberculosis/transmission”[Mesh])) AND (“Homeless Persons/epidemiology”[Mesh] OR “Homeless Persons/mortality”[Mesh] OR “Homeless Persons/statistics and numerical data”[Mesh])*	104
Lilacs http://lilacs.bvsalud.org/en/	*tuberculose [Descritor de assunto] AND pessoas em situação de rua [Descritor de assunto]*	13
Lilacs http://lilacs.bvsalud.org/en/	*pessoas em situação de rua [Descritor de assunto] AND vulnerabilidade social [Descritor de assunto],*	
Lilacs http://lilacs.bvsalud.org/en/	*pessoas em situação de rua [Descritor de assunto] AND vulnerabilidade social [Descritor de assunto] AND tuberculose [Descritor de assunto]*	1
Lilacs http://lilacs.bvsalud.org/en/	*vulnerabilidade social [Descritor de assunto] AND tuberculose [Descritor de assunto]*	5
Lilacs http://lilacs.bvsalud.org/en/	*vulnerabilidade em saúde [Descritor de assunto] AND tuberculose [Descritor de assunto]*	5

**Tabela 2 t2002:** Descritores e artigos selecionados entre as publicações.

Autores	População de estudo	Ano	País	Descritores	Desenho de estudo
Aldridge et al.^21^	491 pessoas em situação de rua	2018	Inglaterra	Tuberculose + Pessoas em Situação de rua	Estudo transversal
Semunigus et al.^22^	351 pessoas em situação de rua	2016	Etiópia	Tuberculose + Pessoas em Situação de rua + HIV	Estudo transversal
Vieira et al.^23^	População de 18 distritos portugueses	2018	Portugal	Tuberculose + Pessoas em Situação de rua	Estudo ecológico
Ranzani et al.^24^	1.726 pessoas em situação de rua	2016	Brasil	Tuberculose + Pessoas em Situação de rua	Estudo de coorte
Hwang et al.^25^	3.292 pessoas em situação de rua	2017	Coreia do Sul	Tuberculose + Pessoas em Situação de rua	Estudo de coorte
Dias et al.^26^	92.053 pessoas em situação de rua	2017	Portugal	Tuberculose + Pessoas em Situação de rua	Estudo de coorte
Agarwal et al.^27^	543 pessoas em situação de rua	2019	EUA	Tuberculose + Pessoas em Situação de rua	Estudo transversal
Nwana et al.^28^	393 pessoas em situação de rua	2019	Estados Unidos	Tuberculose + Pessoas em Situação de rua	Estudo de coorte
Amiri et al.^29^	593 pessoas em situação de rua	2014	Irã	Tuberculose + Pessoas em Situação de rua	Estudo transversal
Powell et al.^30^	110 ocorrências de tuberculose multirresistente	2017	EUA	Tuberculose + Pessoas em Situação de rua	Estudo transversal
Gomez et al.^31^	544 pessoas em situação de rua positivas para tuberculose	2019	Colômbia	Tuberculose + Pessoas em Situação de rua	Estudo de coorte
Munn et al.^32^	64 pessoas em situação de rua	2015	EUA	Tuberculose + Pessoas em Situação de rua	Estudo de coorte
Streit et al.^33^	142 pessoas em situação de rua	2019	Alemanha	Tuberculose + Pessoas em Situação de rua	Estudo transversal
Dolla et al.^34^	301 pessoas em situação de rua	2017	Índia	Tuberculose + Pessoas em Situação de rua	Estudo transversal
Korzeniewska-Koseła et al.^35^	2.349 pessoas em situação de rua	2015	Polônia	Tuberculose + Pessoas em Situação de rua	Estudo de coorte
Pendzich et al.^36^	117 pessoas em situação de rua	2015	Polônia	Tuberculose + Pessoas em Situação de rua	Estudo de coorte

### Critérios de Elegibilidade

Nas análises foram incluídas as publicações de delineamento quantitativo que abordavam a tuberculose e tuberculose+HIV e os fatores de vulnerabilidade associados aos agravos nos idiomas inglês, espanhol e português, publicados entre 2014 e 2020.

### Análise de Viés

A análise de viés das publicações analisadas foi realizada por par e com o uso da ferramenta *Joanna Briggs Institute Critical Appraisal (JBI – Systematic Rewiews tools)* . O JBI é composto por perguntas que avaliam a qualidade metodológica do estudo segundo seu delineamento. Os estudos transversais foram avaliados com o *JBI Critical Appraisal Checklist for Analytical Cross Sectional Studies* com as perguntas: (i) critérios de inclusão e exclusão claramente definidos; (ii) temática e método descritos em detalhes; (iii) exposição mensurada de forma apropriada; (iv) critérios de definição objetivos e padronizados para determinar a condição estudada; (v) identificação de fatores de confundimento; (vi) estratégias para lidar com fatores de confundimento; (vii) desfecho mensurado de forma apropriada; (viii) análise estatística apropriada^18^. Para as análises dos estudos de coorte, utilizou-se o *JBI Critical Appraisal Checklist for Analytical Cohort Studies* , com 11 perguntas: (i) os dois grupos recrutados da mesma população; (ii) as exposições medidas de forma similar para atribuir os dois grupos, expostos e não expostos; (iii) exposição mensurada de forma apropriada; (iv) identificação de fatores de confundimento; (v) estratégias para lidar com fatores de confundimento; (vi) os participantes estavam livres do desfecho no início do estudo; (vii) desfecho mensurado de forma apropriada; (viii) o tempo de estudo suficiente a ocorrência do desfecho; (ix) acompanhamento completo/se não, suas razões descritas e exploradas; (x) estratégias para lidar com acompanhamento incompleto; (xi) análise estatística apropriada^19^. Os estudos ecológicos foram avaliados pelo *JBI Critical Appraisal Checklist for Analytical Cross Sectional Studies* modificado segundo critérios propostos por Dufault e Klar^20^, que descrevem a avaliação metodológica para esse tipo de desenho de estudo, com as seguintes perguntas: (i) explicação sobre delineamento e tamanho amostral escolhido; (ii) critérios de inclusão e exclusão claramente definidos; (iii) temática e método descritos em detalhes; (iv) critérios de definição objetivos e padronizados para determinar a condição estudada; (v) exposição mensurada de forma apropriada; (vi) identificação de fatores de confundimento; (vii) estratégias para lidar com fatores de confundimento; (viii) desfecho mensurado de forma apropriada; (ix) esforços para reduzir possibilidade de viés; (x) análise estatística apropriada; (xi) estratégias para lidar com acompanhamento incompleto; (xii) limitações do estudo apontadas^18 , 20^.

## RESULTADOS

Foram encontradas 374 produções nas plataformas pesquisadas, sendo selecionadas 103 de publicações dentro do período do estudo, das quais duas foram excluídas por serem documentos governamentais oficiais para políticas públicas, 23 foram descartadas por se apresentarem em duplicidade e nove por não estarem indexadas para leitura integral. Ao final, restaram 71 publicações para análise por meio da leitura dos resumos e leitura dinâmica, dos quais foram selecionados 16 segundo os critérios de elegibilidade. A maioria dos estudos se deu entre países do continente europeu (6/16), seguido pela Ásia (3/16), América do Norte (4/16), América do Sul (2/16), com uma publicação brasileira, e África (1/16) ( Figura ). Em relação ao delineamento, sete dos estudos quantitativos eram transversais, oito de coorte e um ecológico. Complementamos os resultados com a média de 2,7 publicações anuais, que se manteve entre os anos analisados, com o mínimo de um e o máximo de quatro trabalhos publicados em cada ano.

**Figura:** Fluxograma de resultados.

A coleta das publicações foi realizada entre junho de 2019 e abril de 2021 e todos os dados foram digitados em planilha *Microsoft Excel* contendo os segmentos: autores, população do estudo, ano de publicação, país de publicação e desenho de estudo.

Entre os artigos selecionados, identificamos os seguintes fatores de vulnerabilidade para os desfechos: uso de álcool^21^, tabagismo^21 , 22 , 25 , 28 , 29^, uso de drogas ilícitas^21 , 24 , 26^, entre elas, injetáveis^21 , 27^, podendo haver compartilhamento de agulhas^29 , 30^, e metanfetamina^29^, histórico de encarceramento^21 , 29^, imigração^26 , 32 , 33^, distúrbio psicológico^24 , 28 , 30^, prostituição^29^, inclusive entre homens que fizeram sexo com homens, analfabetismo^22 , 29^, desnutrição^22^, e coinfecção por HIV^21 , 22 , 24^e outras doenças crônicas como diabetes^27 , 28^, insuficiência renal crônica^27 , 28^e hepatites B e C^21 , 28 , 29^.

Em todos os estudos analisados, a maioria dos pacientes acometidos pela tuberculose era da raça/cor preta ou parda^24 , 28 , 30 , 32^, do gênero masculino e com de idade média de 49,8 anos (DP ± 5,2) e a média de tempo em situação de rua está relacionada à maior ocorrência de tuberculose e infecção latente de tuberculose segundo literatura^21 , 22 , 29^.

Os desfechos relacionados à ocorrência da tuberculose e infecção latente de tuberculose são apresentados na tabela 3 [Table t4002] . A não completude do tratamento^22 , 24 , 25 , 27 , 28 , 30 , 31 , 34 , 35^ou seu fracasso^22 , 24 , 26^, o óbito^22 , 24 , 25 , 27 , 30 , 31 , 34^, desenvolvimento de resistência aos fármacos de tratamento^22 , 25^e ocorrência de tuberculose extrapulmonar^22 , 26 , 27^. Os principais meios diagnósticos foram citologia de escarro^22 , 24 , 26 , 27 , 29 , 31^, R-X torácico^24 , 27 , 33^, cultura^27 , 29^, exame molecular^27^e diagnóstico clínico^27^.

**Tabela 3:** Sumarização dos fatores de vulnerabilidade.

Fator de vulnerabilidade	Dimensão de vulnerabilidade	Autor
Distúrbio psicológico	Individual	Ranzani et al.^24^(2016); Nwana et al.^28^(2019); Powell et al.^30^(2017)
Uso de álcool	Individual e social	Aldridge et al.^21^(2018); Semunigus et al.^22^(2016); Vieira et al.^23^(2018); Ranzani et al.^24^(2016); Hwuang et al.^25^(2017); Dias et al.^26^(2017); Agarwal et al.^27^(2019); Nwana et al.^28^(2019)
Tabagismo	Individual e social	Aldridge et al.^21^(2018); Semunigus et al.^22^(2016); Hwuang et al.^25^(2017); Nwana et al.^28^(2019) Amiri et al.^29^(2014)
Uso de drogas	Individual e social	Aldridge et al.^21^(2018); Ranzani et al.^24^(2016); Dias et al.^26^(2017); Agarwal et al.^27^(2019); Nwana et al.^28^(2019); Powell et al.^30^(2017)
Baixa escolaridade	Individual e social	Semunigus et al.^22^(2016); Ranzani et al.^24^(2016); Amiri et al.^29^(2014)
Imigração	Social	Dias et al.^26^(2017); Munn et al.^32^(2015); Streit et al.^33^(2019)
Encarceramento	Social	Aldridge et al.^21^(2018); Amiri et al.^29^(2014); Powell et al.^30^(2017)
Atuação em trabalhos ilegais (roubo, furto, tráfico de drogas e prostituição)	Social	Amiri et al.^29^(2014)
Desnutrição	Social	Semunigus et al.^22^(2016)
Coinfecção por HIV e outras doenças como hipertensão arterial, hepatites e sífilis, além de recorrência da tuberculose	Social	Aldridge et al.^21^(2018); Semunigus et al.^22^(2016); Ranzani et al.^24^(2016); Hwuang et al.^25^(2017); Dias et al.^26^(2017); Agarwal et al.^27^(2019); Nwana et al.^28^(2019); Amiri et al.^29^(2014)
Pardos e negros	Social	Powell et al.^30^(2017); Munn et al.^32^(2015); Dolla et al.^34^(2017)
Falha no tratamento	Programática	Semunigus et al.^22^(2016); Ranzani et al.^24^(2016); Hwuang et al.^25^(2017); Dias et al.^26^(2017); Agarwal et al.^27^(2019); Nwana et al.^28^(2019); Gomez et al.^31^(2019); Streit et al.^33^(2019); Dolla et al.^34^(2017); Korzeniewska-Koseła et al.^35^(2015)

**Tabela 4 t4002:** Incidências e prevalências achadas nos estudos revisados.

Autores (ano)	Incidência para tuberculose	Prevalência para tuberculose	Prevalência infecção latente de tuberculose	Prevalência tuberculose+HIV	Prevalência infecção latente de tuberculose+HIV
Aldridge et al.^21^(2018)			16,50%		
Semunigus et al.^22^(2016)	505/100 mil hab	2,60%		55,50%	
Ranzani et al.^24^(2016)		2,80%		17,30%	
Hwuang et al.^25^(2017)				5,70%	
Dias et al.^26^(2017)	122/100 mil hab			32,60%	
Agarwal et al.^27^(2019)		4,10%		16%	
Nwana et al.^28^(2019)					1,80%
Amiri et al.^29^(2014)			46,70%		1,20%
Gomez et al.^31^(2019)				20,60%	
Streit et al.^33^(2019			16%		
Dolla et al.^34^(2017)	270/100 mil hab			20%	

Entre as 16 publicações analisadas, em 10 foi descrita a associação entre as comorbidades tuberculose e HIV. Na produção de Semunigus et al.^22^, a proporção de coinfecção chegou a 55,5%. Segundo Dias et al.^26^, ser HIV positivo se mostrou 2,1 vezes mais provável ter um desfecho desfavorável e a comorbidade estava presente em 32,6% dos pacientes analisados. Para Ranzani et al.^24^17,3% das pessoas em situação de rua eram HIV positivas. Segundo Agarwal et al.^27^16% dos participantes com tuberculose eram HIV positivos e a razão de chance para mortalidade nesses indivíduos foi 3.57 maior do que para os com exame de HIV negativo. Na publicação de Hwang et al.^25^5,7% tinha coinfecção tuberculose+HIV. No estudo de Amiri et al.^29^1,2% das PSR eram HIV positivos e tinham infecção latente de tuberculose, proporção similar (1,8%) ao achado por Nwana et al.^28^Gomez et al.^31^, em seu estudo, achou a proporção de 20,6% de PSR com as comorbidades. Vieira et al.^23^relatam que o aumento de 100 casos da coinfecção por HIV+tuberculose na população em geral leva ao aumento da incidência da tuberculose entre pessoas em situação de rua a 14 casos por 100 mil habitantes. No de Dolla et al.^34^, o único paciente HIV+ em sua amostra, veio a óbito.

Em relação aos vieses encontrados, grande parte dos estudos não apresentou similaridade dos grupos recrutados^24 , 25 , 36^, identificação dos fatores de confundimento^26 , 27 , 29 , 30 , 33^, estratégia para lidar com esses fatores^26 , 27 , 29 , 30 , 32^ou com o acompanhamento incompleto dos participantes^25 , 26 , 36^. Poucos estudos trouxeram suas limitações descritas^24 , 26 , 28 , 29 , 31 , 34^e aqueles que apresentaram tinham falhas na exposição da metodologia e dos resultados^29 , 30 , 32 , 36^.

## DISCUSSÃO

Podemos observar uma recorrência entre os resultados relacionada ao consumo de álcool, tabaco e drogas ilícitas – fatores da vulnerabilidade social a partir do estigma associado ao vício e que também é uma susceptibilidade individual –, seja para a fuga da realidade de sofrimento ou ainda para buscar uma melhoria do bem-estar geral^37 , 38^. Assim, essa sobreposição de vulnerabilidades pode aumentar a exposição à tuberculose e HIV na população do estudo. O sexo e idade, assim como a coinfecção por outras doenças transmissíveis e histórico de encarceramento, também são fatores que despertam discriminação na nossa sociedade, embora em níveis diferentes.

A falta de completude do tratamento e desenvolvimento de resistência aos medicamentos de tratamento estão incluídos na esfera da vulnerabilidade programática, pois demonstram a falha do sistema de saúde em fornecer tratamento, informação e estrutura de forma adequada para o tratamento desses pacientes.

Encontramos uma variabilidade na quantidade de publicações anuais com os temas tuberculose e HIV para a população de PSR. A média anual foi de menos de três (2,67), considerado pouco na comparação com outras populações também classificadas como vulneráveis. Tratando-se de um agravo muito importante de saúde pública mundial, a ponto de estar listada entre os Objetivos do Desenvolvimento Sustentável, chama a atenção algumas falhas metodológicas como: pequeno tamanho da amostra, o que fragiliza os resultados e conclusões apresentados; falta de acesso direto aos pacientes positivos para tuberculose^29^; subestimação do número real de pessoas em situação de rua^24 , 26^; e da incidência e prevalência da tuberculose nessa população^26 , 34^; amostra composta somente de pessoas com tuberculose^24^, excluindo-se as formas extrapulmonares.

### Vulnerabilidade Individual

Entre as publicações revisadas, o uso de drogas ilícitas, álcool ou tabaco que fazem parte dos fatores de vulnerabilidade individual e social apontaram para importância dessas susceptibilidades na vida da PSR, apontando para a necessidade de uma atenção singular desses aspectos no enfrentamento da tuberculose entre a PSR.

O uso constante de drogas pode levar ao desenvolvimento de transtornos mentais, como afirma o último relatório mundial sobre drogas, em 2020, do Escritório de Drogas e Crimes da Organização das Nações Unidas (UNODC). Em duas publicações^24 , 30^foi descrita a variável “transtorno mental”, evidenciando sua constante ocorrência entre as amostras usadas para os estudos. O rompimento com familiares, seja por não adequação ao seu modelo estrutural, seja por histórico de violência e assédio, ou mesmo por não aceitarem formas de sustento escusas e o próprio vício, expõe essas pessoas a uma condição de vulnerabilidade social e individual, pois não podem contar com suas famílias num momento de dificuldade^39 , 40^.

### Vulnerabilidade Social

A redução da situação de vulnerabilidade social é enfrentada pelo Estado com a implantação de programas de transferência de renda pelo governo, que são artifícios para diminuir o número de pessoas abaixo da linha da pobreza e dar a elas melhores condições de alimentação^41^. Essa importante medida é considerada programática, com consequências sobre a vulnerabilidade social e deve ser ampliada, pois pode reduzir a vulnerabilidade das pessoas na linha abaixo da miséria. A sobreposição ou intersecção das dimensões de vulnerabilidade ajudam a ampliar o olhar da epidemiologia para a forma de adoecer e alertar os gestores para as questões de saúde oportunizando um enfrentamento mais qualificado desses problemas entre PSR.

A produção de vidas vulnerabilizadas e não integráveis faz com que as pessoas em situação de rua convivam continuamente com a dificuldade no acesso à educação, trabalho, cuidado e serviços de saúde, entre outros, além da sua invisibilidade, o que pode levar algumas dessas pessoas ao envolvimento em atividades como prostituição, associação ao tráfico, roubo e furto e trabalho análogo à escravidão.

A vulnerabilidade social também envolve características marcadas pela classe social baixa, local de moradia, raça negra, entre outras. A proporção de pretos e pardos entre os resultados encontrados nas publicações foi de 63,6%. A idade média dos participantes dos estudos é de 49,8 anos (DP ± 5,2), provavelmente devido à maior mobilidade espacial se comparado às mulheres ou pessoas de diferentes faixas etárias, como idosos, que transitam menos pelas ruas, se expondo menos em seus trajetos^42^.

O histórico de encarceramento foi frequente entre os estudos usados nessa revisão. O sistema prisional, incumbido de reabilitar e reinserir o apenado à sociedade, falha nessa tentativa de reinserção devido ao estigma que o condenado carrega após o cumprimento de sua pena, seja pela sociedade em geral, pela falta de apoio familiar ou pela falta de oportunidade de emprego, o que faz com que muitos acabem sem recursos monetários para continuarem sua vida e tenham que recorrer à vida nas ruas, algumas vezes atuando em trabalhos ilegais, o que não rompe o ciclo de reincidência^43^.

Segundo *ranking* realizado pelo *World Prison Brief* (2021)^44^, o Brasil tem a terceira maior população carcerária do mundo, fato intrinsecamente ligado ao racismo estrutural e, consequentemente, à marginalização sistemática de pessoas pretas e pardas, compreendendo 61,7% dos encarcerados^45^. Com isso, podemos observar que esses fatores de vulnerabilidade relacionados ao recorte social, racial e econômico se perpetuam nas populações mais precárizadas.

### Vulnerabilidade Programática

Essa dimensão da vulnerabilidade, relacionada ao acesso aos equipamentos públicos e institucionais do Estado, revelou-se uma importante dimensão da análise das susceptibilidades de populações vulnerabilizadas em relação à tuberculose e ao HIV, nos estudos epidemiológicos^46^, pois fornece evidências da precariedade e fragilidade desses recursos. A falta de completude do tratamento e desenvolvimento de resistência aos medicamentos de tratamento estão incluídos nessa dimensão, pois demonstram a falha do sistema de saúde em fornecer tratamento, informação e estrutura adequados para o tratamento desses pacientes^47^. Entre as consequências está a coinfecção com outras doenças, visto que a habitação nas ruas e sua exposição ambiental potencializam os riscos para algumas doenças crônicas e infecciosas^38^, pois questões prioritárias à manutenção da vida, como segurança e alimentação, são mais urgentes que o cuidado com a saúde, e diante das dificuldades de acesso, o cuidado com a saúde fica em segundo plano ou quando surgir uma oportunidade^38 , 48^.

## CONCLUSÃO

O Brasil, segundo o Instituto de Pesquisa Econômica Aplicada, contava com mais de 100 mil pessoas vivendo nas ruas em 2016^49^. Atualmente, diante das perspectivas socioeconômicas brasileiras, inclusive no contexto da pandemia da covid-19 aliado ao cenário econômico e político, já se observa um aumento da pobreza e na quantidade da PSR^50^. Entre as consequências é possível que se observe a curto prazo um aumento da tuberculose na PSR, como também em outras populações vulnerabilizadas, a exemplo das populações de baixa renda, moradores de favelas ou ainda nos moradores de habitação precária^51^, visto que já existe um panorama sobre o aumento da pobreza e miséria no Brasil, assim como em outros países da América Latina^50 , 52^.

Nosso olhar pela perspectiva do conceito de vulnerabilidade permitiu verificar que os resultados encontrados nas publicações analisadas evidenciaram a questão social e programática dos fatores associados à tuberculose e HIV entre PSR, destacando as diferentes dimensões da precarização das vidas e atenção a sua saúde. Assim, esse conceito foi um suporte fundamental para classificar e entender os diferentes tipos de susceptibilidades que permeiam a vida das PSR e dificultam o tratamento da tuberculose e HIV, bem como falha no tratamento por descontinuidade e, consequentemente, aparecimento de cepas resistentes.

Mesmo com protocolos e diretrizes estabelecidas, portarias e normas técnicas para a prevenção e tratamento dos agravos aqui abordados para a PSR, percebeu-se que a execução dos planos de ações traçados pelas estratégias governamentais apresenta importantes falhas, evidenciando as fragilidades do Sistema de Saúde e políticas públicas voltadas para PSR, bem como as questões de estigma e preconceitos sociais que se reproduzem nos serviços de saúde. Dessa forma, algumas vidas específicas estão mais expostas a situações de vulnerabilidade e, consequentemente, com menos acesso a prevenção e proteção, logo, mais susceptíveis à violência do Estado e da sociedade do que outras. Isso é produzido por variados atravessamentos que marcam essas vidas como mais vulneráveis e muitas vezes não reconhecidas, sendo invisibilizados.

### Recomendações

A partir dos resultados dessa revisão, recomenda-se uma maior atenção e investimento em melhoria das ações de prevenção e intervenções para a tuberculose e tuberculose+HIV na PSR, desde a atenção básica, mantendo uma rotina de avaliação das vulnerabilidades das pessoas em situação de rua. Aos gestores e técnicos, avaliar a necessidade de alteração e ajustes dos protocolos da atenção básica para as PSR, além da distribuição integral de recursos para as demandas já conhecidas e aqui apresentadas. Também é necessário esforço e comprometimento político com a distribuição dos recursos destinados à saúde, políticas sociais e de assistência as PSR.

A sistematização dos fatores de vulnerabilidade pode ser utilizada para embasar hipóteses para futuras pesquisas, subsidiar políticas públicas de saúde e sociais para o enfrentamento da tuberculose e HIV na PSR. Os achados, quando comparados a outras revisões sistemáticas, parecem avançar ao discutir pela ótica do conceito de vulnerabilidade, o que é inédito nas publicações nacionais, para a população das pessoas em situação de rua no contexto da tuberculose e tuberculose+HIV.

### Limitações

Esse estudo apresenta limitações, visto que mesmo que realizada uma ampla busca de trabalhos publicados por duas pesquisadoras, uma quantidade de textos pode ainda não estar disponível de forma digital nas plataformas e, devido à velocidade da dinâmica das publicações, alguns trabalhos podem ter escapado à busca e coleta de dados.
